# Stability of Chlorophyll *a* Monomer Incorporated into Cremophor EL Nano-Micelles under Dark and Moderate Light Conditions

**DOI:** 10.3390/molecules25215059

**Published:** 2020-10-30

**Authors:** Ewa Janik-Zabrotowicz, Marta Arczewska, Patrycja Prochniewicz, Izabela Świetlicka, Konrad Terpiłowski

**Affiliations:** 1 Department of Cell Biology, Institute of Biological Sciences, Maria Curie-Sklodowska University, Akademicka 19, 20–033 Lublin, Poland; patrycja.prochniewicz@wp.pl; 2 Department of Biophysics, University of Life Sciences in Lublin, Akademicka 13, 20–950 Lublin, Poland; izabela.swietlicka@up.lublin.pl; 3 Department of Physical Chemistry-Interfacial Phenomena, Maria Curie-Sklodowska University, 3, 20–031 Lublin, Poland; terpil@poczta.umcs.lublin.pl

**Keywords:** Cremophor EL, chlorophyll monomer, molecular spectroscopy, photostability

## Abstract

In this paper, stability of chlorophyll *a* monomers encapsulated into the Cremophor EL nano-micelles was studied under dark and moderate light conditions, typical of a room with natural or artificial lighting, in the presence of oxygen. The pigment stability against visible light was determined using the dynamic light scattering and molecular spectroscopy (UV-Vis absorption and stationary fluorescence) methods. Chlorophyll *a*, at the molar concentration of 10^−5^ M, was dissolved in the 5 wt% Cremophor emulsion for comparison in the ethanolic solution. The stability of such a self-assembly pigment–detergent nano-system is important in the light of its application on the commercial-scale. The presented results indicate the high stability of the pigment monomeric molecular organization in the nano-emulsion. During the storage in the dark, the half-lifetime was calculated as about 7 months. Additionally, based on the shape of absorption and fluorescence emission spectra, chlorophyll aggregation in the Cremophor EL aqueous solution along with the time was excluded. Moreover, the average size of detergent micelles as chlorophyll carriers was not affected after 70 days of the nano-system storage. Pigment stability against the moderate white light (0.1 mW) did not differ significantly from storage conditions in the dark. The photooxidation products, detected by occurrence of new absorption and fluorescence emission bands, was estimated on the negligible level. The stability of such a self-assembly pigment–detergent nano-system would potentially broaden the field of chlorophyll *a* (chl *a*) application in the food industry, medicine or artificial photosynthesis models.

## 1. Introduction

Chlorophylls (chlorophyll *a*-*f*) and bacteriochlorophylls are well-known, non-toxic, and the only natural green pigments. Chlorophyll *a* (chl *a*) has existed on the Earth for at least 2.6 billion years. It is estimated that its annual production is 1.2 billion tons worldwide [1]. In the photosynthetic organisms (higher plants, some bacteria, and algae), it is bound to the protein bed of the photosynthetic complexes in the thylakoid membranes of chloroplasts. This pigment is responsible for the absorption of blue and red sunlight being the best photoreceptor in nature. This pigment ability is then used in the light phase of photosynthesis providing life on the Earth.

Chl *a* can be extracted from plants using suitable solvents. However, in the solutions, chl *a* is highly degraded by light, heat, acids, and enzymes [1]. Moreover, it is highly unstable during storage and processing, resulting in color changes in naturally colored food [1]. Loss of green color is mostly caused by chl pheophytinization. On the other hand, numerous investigations are dealing with chl *a* practical applications. It is applied as a natural colorant of food [2,3]. Moreover, it has been used in cosmetology (e.g., against acne vulgaris [4]). This biomolecule possesses anti-cancer [5,6,7,8], anti-bacterial, anti-inflammatory, anti-proliferative, deodorizing, and wound healing activities [1]. Unfortunately, chl *a* is a hydrophobic molecule, and in water media, it is characterized by low solubility resulting in reduced bioavailability and cellular uptake. As a result of these limitations, various chl derivatives are applied on the commercial scale. Sodium/copper chlorophyllin is the most common, widely used copper–chlorophyll derivative. This complex is characterized by better solubility and stability in water compared with chl [9]. In China and Europe, it is approved for use, but in the US its application is limited due to safety issues [3]. Moreover, the word “chlorophyll” is often incorrectly used as a description for many commercial products containing chlorophyllin, giving rise to some confusion. Among 30 commercial products (cosmetics and supplements of a diet containing a green pigment) selected by us, only 6 appropriate descriptions (natural chlorophyll or chlorophyllin, respectively) were given. So, it is required to find a molecular form of chl that is soluble and stable in an aqueous medium safe for health.

In this context, recently it has been reported that chl *a* dissolves more efficiently in the aqueous medium containing the low-level (5 wt%) non-ionic detergent—Cremophor EL (CrEL) than in an ethanolic solution [10]. Cremophor EL (polyoxyl 35 castor oil) is a polyoxyethylated derivative of hydrogenated castor oil that contains about 87% of ricinoleic acid. CrEL nano-emulsion is attractive because of its remarkable small micelles size (about several nanometers) and thermodynamic stability. This nano-emulsion attracted much attention in the pharmaceutical applications for encapsulation and solubilization of various hydrophobic bioactive compounds [11,12,13,14,15]. The results have shown that the molecular organization of chl *a* in the CrEL nano-emulsion is monomeric [10]. This is relevant due to the high photoactivity of chl monomer [16] and the expected efficient uptake of such a small molecule but not a larger aggregated form.

Thus, this paper is focused on evaluating the stability of chl *a* monomers in the Cremophor EL nano-micelles under the dark and moderate light conditions. The aqueous solution containing chl *a* in 5 wt% CrEL was monitored during storage using UV-Vis absorption and stationary fluorescence spectroscopy.

## 2. Results and Discussion

**Figure 1:**
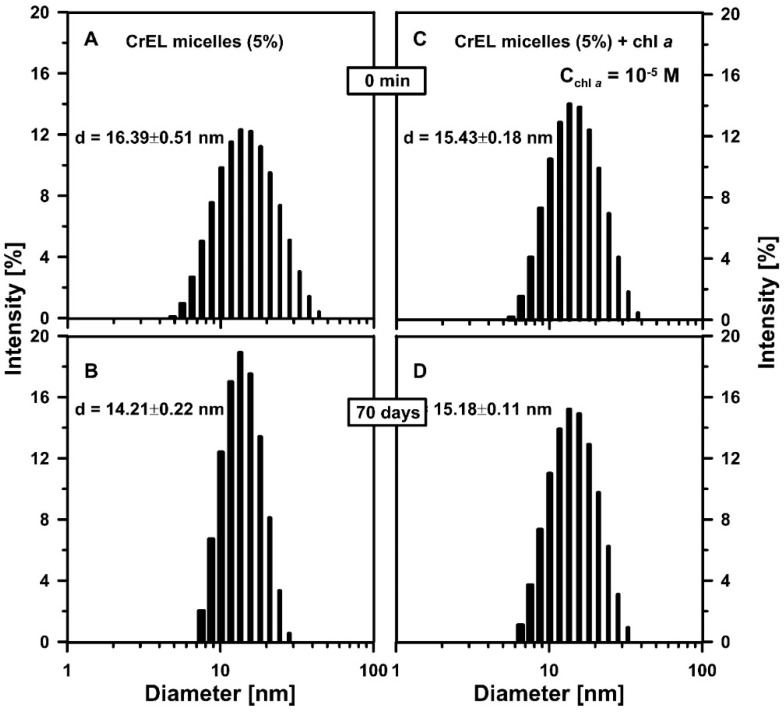
Cremophor micelles size distribution histograms. (**A**,**B**) the PBS buffer containing 5 wt% Cremophor EL (CrEL) (CrEL micelles (5%), 5 wt% Cremophor nano-emulsion). (**C**,**D**) An amount of 5 wt% Cremophor nano-emulsion mixed with chlorophyll *a* (chl *a*) at the molar concentration of 10^−5^ M (CrEL micelles (5%) + chl *a*). (**A**,**C**) The histograms were measured directly after the sample preparation (0 min). (**B**,**D**) The samples were stored for 70 days in the dark conditions. Histograms were obtained using the dynamic light scattering method. The representative sizes of nano-micelles are included in the figure (obtained from the presented histograms, numbers represent the mean values obtained by replicate measurements of the same sample, *n* = 3 technical replicates). The experiment was repeated three times (biological replicates).

As it was proved earlier [10], chl *a*, at the molar concentration of 10^−5^ M, is monomeric in the aqueous medium containing low-level (5 wt%) CrEL. As the next step of these studies, the stability of the green pigment–detergent nano-system was tested. The long-term stability of chl *a* monomeric form under the oxygenic conditions is an especially important feature in this system application on the commercial scale. Firstly, the application of the dynamic light scattering method to determine the stability of the average size of CrEL micelles as chl *a* carriers after 70 days of storage at room temperature in the dark conditions was studied (Figure 1). The CrEL micelles in the 5 wt% emulsion were about 16 nm in size (Figure 1A). This average size did not even change significantly after 70 days (Figure 1B). It is an important factor because micelles must remain intact during drug formulation and storage. Moreover, the diameter of micelles with incorporated chl *a* was also stable over time (~15 nm, Figure 1C,D).

**Figure 2:**
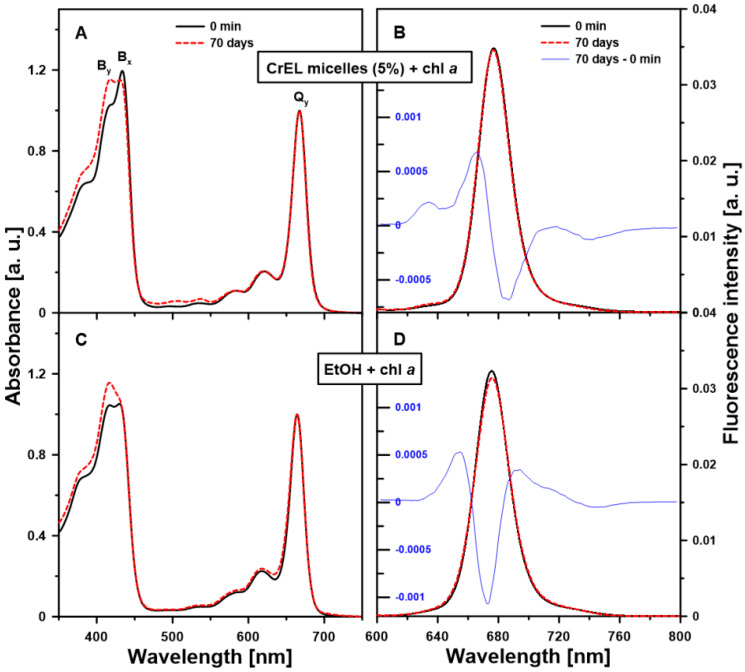
Room temperature electronic absorption and fluorescence emission spectra measured from chl *a* dissolved in the 5% Cremophor EL nano-emulsion (CrEL micelles (5%) + chl *a*; panels (**A**,**B**)) or 96% ethanol (EtOH+ chla *a*; panels (**C**,**D**)). The molar concentration of chl *a* in the samples was 10^−5^ M. The spectra were registered directly after the sample preparation (0 min, black solid line) and after 70 days of storage of the samples in the dark (70 days, red dashed line). The experiment was repeated three times (biological replicates). The representative results are included in the figure. The absorbance spectra were normalized in the Q_y_ band and the fluorescence emission spectra were normalized to get the same area beneath each spectrum. In (**B**,**D**) panels, the difference spectra were added (70 days–0 min, blue thin line). The emission spectra were registered with the excitation at 434 nm.

Since chl *a* tends to precipitate in water and destabilize under the oxygen conditions, the stability of chl *a* monomeric molecular form in the aqueous medium containing low-level (5 wt%) CrEL was monitored during storage using the simplest and most useful methods to study chl optical properties and therefore its molecular structure: UV-Vis absorption and stationary fluorescence spectroscopy. In Figure 2, the electronic absorption (Figure 2A) and fluorescence emission (Figure 2B) spectra of chl *a* dissolved in the CrEL nano-emulsion are presented. To compare, chl *a* was also dissolved in 96% EtOH (Figure 2C,D) because chl *a* at the 10^−5^ M molar concentration in 96% EtOH should also be monomeric [10]. Moreover, EtOH is an important organic solvent extensively used in the food, pharmaceutical, and cosmetic industries.

**Figure 3:**
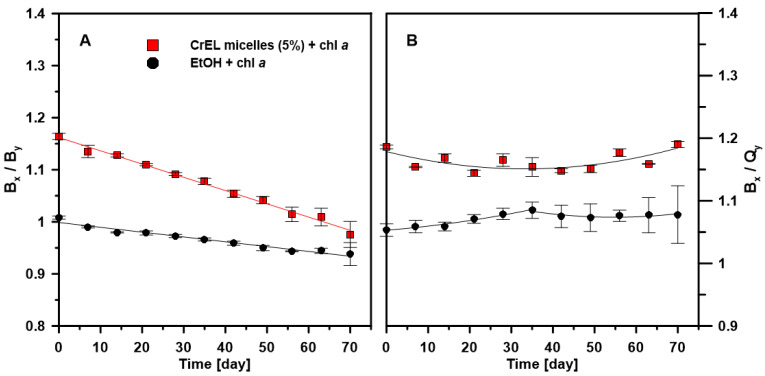
Stability of chl *a* in the CrEL nano-micelles over time. Stability was expressed as B_x_/B_y_ (panel **A**) and B_x_/Q_y_ (panel **B**) ratios varying with time (70 days). Chl *a* at the molar concentration of 10^−5^ M was dissolved in the 5% Cremophor EL nano-emulsion (CrEL micelles (5%) + chl *a*; red squares) or, for comparison, in 96% ethanol (EtOH, black circles). The samples were stored in the dark for 70 days. The experiment was repeated three times (biological replicates). The presented mean values ±SD are obtained on the basis of the absorbance spectra.

As can be seen in Figure 2, the main absorption maxima of chl *a* dissolved in the nano-emulsion are located at 434 nm and 417 nm (Soret spectral region, B_x_ and B_y_ bands) and 668 nm (Q spectral region, Q_y_ band). These bands for chl *a* in the EtOH solution are located at 430 nm, 417 nm, and 663 nm, respectively. Soret and Q spectral bands arise from π → π* transitions of the four frontier orbitals [17]. One band of each pair is polarized along the x-axis (B_x_, Q_x_) and the other one along the y-axis (B_y_, Q_y_). The y-axis is defined as passing through the N atoms of rings A and C and the x-axis as passing through the N atoms of rings B and D [18] in the porphyrin ring. The size and shape of the π electron system are the major determinants of the optical properties of chls [19,20]. The fluorescence emission spectrum has a maximum at 677 nm for chl *a* dissolved in the CrEL emulsion (Figure 2B) and at 675 nm for chl *a* dissolved in EtOH (Figure 2D). The position of the bands in the absorption and fluorescence emission spectra points out to the chl *a* monomerization in the solutions [10]. Thus, the question of whether “If the monomeric form of chl *a* is still present in the CrEL nano-emulsion over time” is addressed in this paper. As the changes in the intensity of the whole absorption and fluorescence emission spectra may additionally arise from chl *a* molecular reorganization caused by evaporation of the sample solvent (the effect mainly visible in the case of the ethanolic solution, Figure S1), the molecular organization of chl *a* was only checked based on the spectral shape (absorbance spectra were normalized in the Q_y_ band, fluorescence spectra were normalized to get the same area beneath each spectrum). If the chl *a* aggregation took place during storage over a long period of time, the Q_y_ band should be red-shifted and a new, low energy absorption band (≥700 nm) should appear. This feature is visible in the absorption spectrum of chl *a* aggregates in the PBS buffer (Figure S2). As can be seen in Figure 2A,C, the shape and position of Q_y_ band of chl *a*, dissolved in both the nano-emulsion or EtOH solution, were not changed after 70 days. So it can be concluded that the molecular symmetry and the macrocycle saturation, which are closely related to the Q_y_ energy, were not time-affected [19]. However, the intensity of B_y_ absorption band is negligibly increased with the B_x_ band intensity after 70 days in the solutions. Moreover, the relation between the B_x_/B_y_ ratio and the time of storage was determined (Figure 3).

The decreasing pattern in the value of B_x_/B_y_ ratio for chl *a* embedded in the CrEL micelles is similar to chl *a* dissolved in EtOH. The value of B_x_/B_y_ ratio decreased linearly only by 13% and 9% for chl *a* in the CrEL micelles and EtOH, respectively. This phenomenon may suggest changes in the pigment monomer structure related to the electronic transition along the y-axis. Importantly, the ability to absorb the blue and red light by chl *a* expressed as the ratio of the main absorption maxima intensity (B_x_/Q_y_) (Figure 3B) did not vary significantly after 70 days for both chl *a* dispersed in the CrEL nano-emulsion and EtOH.

Chls are the most stable in their natural environment of the photosynthetic complexes. For example, Mattila and co-workers have proven that in the majority of trees, the chlorophyll content in the leaves remained stable until a phase of rapid pigment degradation caused by the autumn senescence [21]. As chls degrade and discolor readily beyond nature (mainly in the presence of oxygen), their stability and degradation in the artificial systems have been a subject of interest for many scientific studies. In such an environment as water [10,22], the mixture of water and EtOH [10,22,23], PVA films [24,25], polymeric nanomaterials [5,26], mesoporous materials [27], silica gel [27,28], chl *a* is aggregated. It was proved that chl *a* aggregates remain stable in such systems for few months. However, photostability or stability in the dark of chl *a* monomers in EtOH [19,25], toluene [29], benzene [27], chloroform [30], Triton X-100 [31] is less than one day. On the other hand, about 50% of the monomers pool inserted in the biomimetic membranes was stable in the period of 9 months in the presence of oxidative stress [32]. Hence, it was very important to determine the half-lifetime (the time during which a 50% decay of Q_y_ band intensity occurs) of chl *a* molecule upon 5 wt% CrEL in water medium. This parameter was determined based on the lowest energy band decay [19]. The changes in a value of Q_y_ band intensity (presented as percentage of the initial value) during storage were fitted into the equation shown in Figure S3 (with the determination coefficient value of 0.90). The half-life time was calculated as 215 days (~7 months). This value is comparable with the half-life time estimated for chl *a* in green beans during frozen storage [33].

To confirm the stability of chl *a* monomers in the studied nano-micelles over time, the fluorescence emission spectra were normalized to get the same area beneath each spectrum (Figure 2B). The shape and position of the fluorescence emission bands provide important information about the molecular organization of the studied pigment because the electronic structure of its macrocycle is strongly affected by the aggregation/disaggregation process. The shape of the spectrum measured after 70 days of storage of the sample differs marginally from that of the spectrum detected directly after the sample preparation. The appearance of a relatively weak emission band at 666 nm in the difference spectrum indicates the hypsochromic shift of the main emission maximum (677 nm). Additionally, the very low fluorescence emission from the spectral form at 635 nm was registered. These results indicate that chl *a* destruction products are present in the samples, and are characterized by a reduced amount of conjugation in their chromophore [30,34]. On the other hand, very low intensity of the described bands indicates a negligible pool of these products which proves a high stability of the chl *a* monomeric form in the nano-emulsion along with time. Based on the difference spectrum regarding the pigment dissolved in EtOH (Figure 2D), chl *a* aggregation after the 70-day storage in this solution was additionally observed. The evidence for this is provided by the formation of the spectral forms with long-wavelength emission bands at 690 nm and 710 nm [35,36,37]. However, it is worth noting that, at room temperature, the quantum yield of the fluorescence of aggregates is very low due to the rapid relaxation. In this way, it is difficult to estimate a real quantity of aggregates in the discussed case.

From the above results, it can be inferred that the nano-micelles made of CrEL detergent in the water medium offers high storage stability of chl *a* monomeric molecular form in the dark and under the air conditions. Such a system may be successfully used instead of chl dissolved in EtOH. This is a very important observation due to the known toxicity of ethanolic solution above 15% for human health [38].

Since chl *a* in the presence of light and oxygen degrades readily and irreversibly, the photostability of chl *a* monomers in 5 the wt% CrEL emulsion was tested by the illumination of such a system with white light for 1 h. Being not a natural light source, a halogen lamp was used to provide stable, controlled conditions of the experiment. Radiation of this lamp covers a large range of the wavelengths emitted by the Sun and the typical artificial light source as a fluorescent bulb (Figure S4). The light power density for the sample was 0.1 mW/cm^2^ or 1 mW/cm^2^. These values were selected from the measurements of light intensities in rooms with natural or artificial lighting, which usually ranges from about 0.1 to 1 mW/cm^2^. Such light regimes are relevant in the context of quality of both long-term storage and the formulations with chl *a* incorporated into the 5 wt% CrEL nano-emulsion under moderate light conditions.

**Figure 4:**
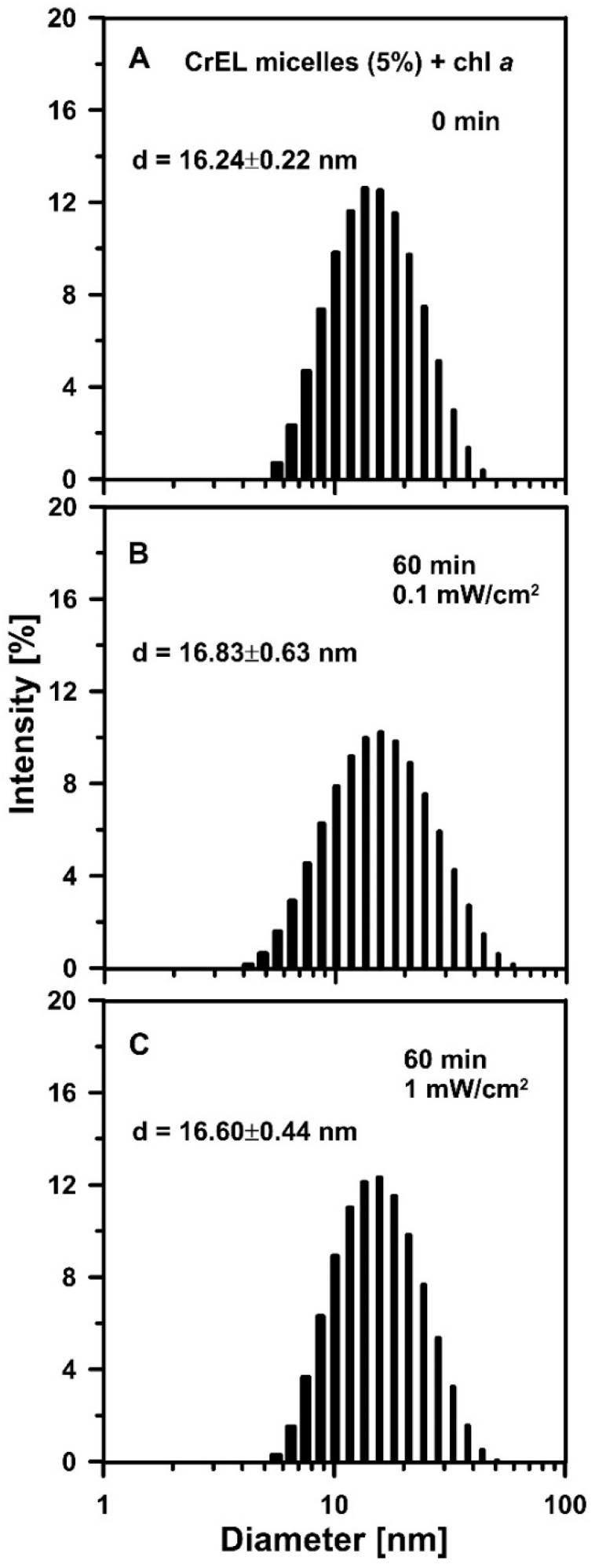
Cremophor micelles size distribution histograms. (**A**) An amount of 5 wt% Cremophor nano-emulsion mixed with chl *a* at the molar concentration of 10^−5^ M (CrEL micelles (5%) + chl *a*). The histogram was measured directly after the sample preparation (0 min). (**B**) The CrEL nano-micelles with chl *a* illuminated with white light for 60 min. The light power was 0.1 mW/cm^2^. (**C**) The CrEL nano-micelles with chl *a* illuminated with white light for 60 min. The light power was 1 mW/cm^2^. Histograms were obtained using the dynamic light scattering method. The representative sizes of nano-micelles are included in the figure (obtained from the presented histograms, numbers represent the mean values obtained by the replicate measurements of the same sample, *n* = 3 technical replicates). The experiment was repeated three times (biological replicates).

As can be seen in Figure 4, with 0.1 mW/cm^2^ and 1 mW/cm^2^ power density applicable for 1 h nano-micelles with the embedded chl *a* monomers exhibited excellent stability of an average diameter of ~16 nm.

**Figure 5:**
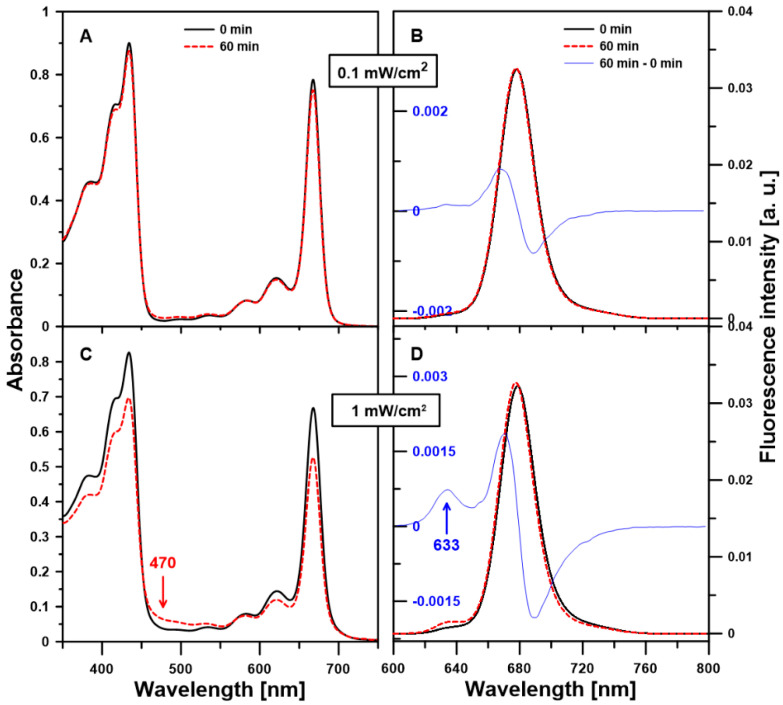
Room temperature electronic absorption and fluorescence emission spectra measured from chl *a* dissolved in the 5% Cremophor EL nano-emulsion. The molar concentration of chl *a* in the samples was 10^−5^ M. The spectra were registered directly after the sample preparation (0 min, black solid line) and after the illumination of the samples with white light for 60 min (red, dashed line). The light power was 0.1 mW/cm^2^ (panels: (**A**,**B**)) or 1 mW/cm^2^ (panels: **C**,**D**). The experiment was repeated three times (biological replicates). The representative results are included in the figure. The fluorescence emission spectra were normalized to get the same area beneath each spectrum. Panels (**B**,**D**) illustrate additionally the difference spectra (60 min–0 min, blue thin line). The emission spectra were registered with excitation at 434 nm.

**Figure 6:**
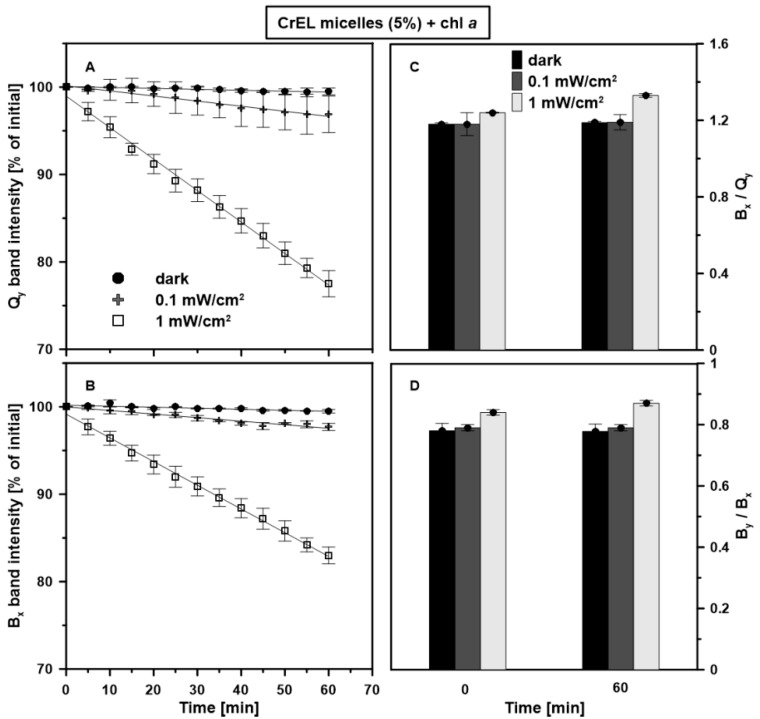
Photostability of chl *a* in the CrEL nano-micelles. Stability was expressed as the Q_y_ and B_x_ bands intensity (panels: **A**,**B**), B_x_/Q_y_ and B_y_/B_x_ ratios (panels: **C**,**D**) varying with illumination for 60 min. The molar concentration of chl *a* was 10^−5^ M. The samples were dark-adapted for 60 min (black circles) or illuminated with white light for 60 min. The power of light was 0.1 mW/cm^2^ (grey crosses) or 1 mW/cm^2^ (white squares). The experiment was repeated three times (biological replicates). The presented mean values ±SD are obtained on the basis of the absorbance spectra.

The photostability of chl *a* monomers in the CrEL micelles was investigated by absorption and fluorescence emission spectroscopy. In Figure 5, the UV-Vis absorption and fluorescence emission spectra of chl *a* monomers in the CrEL nano-micelles measured directly after the samples preparation and after 60-min illumination with visible light (power density of 0.1 or 1 mW/cm^2^) are presented. The dependence of the chl *a* photostability on the irradiation power density is shown in Figure 6. It is expressed as a time course of Q_y_ and B_x_ bands intensity and values of B_x_/Q_y_ and B_y_/B_x_ ratios after the pigment–detergent system illumination for 60 min. As can be seen, chl a is quite stable under light intensity of 0.1 mW/cm^2^. The values of Q_y_ and B_x_ bands intensity decreased linearly only by 2.6% (panel A) and 1.8% (panel B) of control with the continuous visible light. Additionally, during 1 h radiation, no significant differences in the value of B_x_/Q_y_ (panel C) and B_y_/B_x_ (panel D) ratios were found (Table 1, Tables S2 and S3). Moreover, for the studied system, the shape of the fluorescence emission spectrum measured after 1-h illumination remained mostly unchanged (Figure 5B). For determination of the half-life time of chl *a* monomers in the 5 wt% CrEL emulsion at an excitation power density of 0.1 mW/cm^2^, the adequate data presented in Figure 6A were linearly fitted with the determination coefficient value of 0.99. Based on the obtained equation, the half-life time of chl *a* monomers value was calculated as τ_1/2_ = 14 h.

Lower photostability of chl *a* monomers against the light power density of 1 mW/cm^2^ was observed according to the alteration in the intensity and shape of the absorption spectra during illumination. The half-life time of chl *a* value was calculated as τ_1/2_ = 2.3 h. As follows from Figure 6, the intensity of Q_y_ and B_x_ bands changed by 22.1% and 16.6%, respectively, at the end of the sample irradiation. Moreover, the values of B_x_/Q_y_ and B_y_/B_x_ ratios increased by 7.3% and 3.4%, respectively, compared with the control sample. An increase in the B_x_/Q_y_ value suggests changes in the chl *a* molecule structure resulting in more efficient red-light absorption compared to blue light.

However, a small increase in the value of B_y_/B_x_ ratio points out to the decomposition of the porphyrin ring related to the electronic transition along the y-axis. Moreover, the increase in the absorbance intensity at 470 nm in the absorption spectrum (Figure 5C) and fluorescence emission at 633 nm in the emission spectrum (Figure 5D) measured after illumination for 1 h with 1 mW/cm^2^ also indicates that the degradation products have been formed under the light conditions [30,39]. The same new positive bands in the absorbance and fluorescence emission spectrum were observed when chl *a* monomers in the 5 wt% CrEL emulsion were exposed to strong red light (20 mW/cm^2^, Figure S5) in the air for 1 h. Based on the shape of the difference spectrum (Figure 5D), the spectrum measured after 1 h of the sample irradiation minus the spectrum registered directly after the sample preparation), it may be concluded that these degradation products are characterized by a reduced amount of conjugated double bonds in their chromophore (hypsochromic shift of the main emission maximum), derived from the known photooxidation of chl *a*. The light absorption chl *a* molecules can convert into the long-lived triplet excited state. This state reacts with the triplet ground state of oxygen readily generating strongly photo-oxidative reactive oxygen species (singlet oxygen and radical oxygen substances) [29,30,40]. It is important to note that the low intensity of the described new spectral bands may prove the stability of chl *a* monomeric form in the aqueous medium containing low-level (5 wt%) CrEL detergent under moderate light conditions. On the other hand, the presence of photooxidation products in the samples indicates that the studied system would be a promising photosensitizer at light intensities stronger than 1 mW/cm^2^. It would be attractive in the context of chl *a* monomers application because the tendency towards undesirable aggregation characterizes many photosensitizers (e.g., used in photodynamic therapy).

## 3. Materials and Methods

### 3.1. Materials

All used chemicals were of analytical grade. CrEL (polyoxyl 35 castor oil, MW = 2500 g⋅mol^−1^) and phosphate buffered saline (PBS, pH 7.4) were purchased from Sigma Aldrich (Milwaukee, WI, USA). Chl *a* (MW = 893.5 g⋅mol^−1^) was isolated from fresh spinach leaves. The detailed protocol is described in [10]. PBS solutions were prepared with deionized water (not previously degassed) of conductivity ≤0.06 μS/cm from a HLP 10 system (HydroLab, Straszyn, Poland). Finally, PBS solutions for each experiment were filtered through a set of membrane filters (Millipore Express Plus, 0.22 μm). The concentration of dissolved oxygen in aqueous solutions was recorded using a HI-2004 Edge^®^ Dissolved Oxygen Meter with a HI 764080 electrode (Hanna Instruments, Woonsocket, RI, USA). All samples were prepared and stored in brown glass vials with screw caps (Thermo Fisher Scientific, San Jose, CA, USA).

#### 3.1.1. Chlorophyll *a* in Ethanol

Firstly, chl a was evaporated from acetone in glass vials. Next, an adequate volume of EtOH was added to the sample. The mixture was slowly stirred at 23 °C for 15 min.

#### 3.1.2. Cremophor Nano-Emulsion Preparation

Firstly, the non-ionic surfactant (CrEL) was dissolved in the co-surfactant (96% EtOH). Next the mixture was added into an aqueous phase (PBS buffer, (pH 7.4)) by slow stirring (500 rpm) at 23 °C for 1 h for spontaneous formation of nano-micelles of CrEL. The final concentration of CrEL was 5 wt%.

#### 3.1.3. Complex Formation with Chlorophyll *a*

Chl *a* was dissolved in 96% EtOH. Next, it was added to the Cremophor emulsion, while slowly stirring (500 rpm) at 23 °C for 15 min at the ratio 1:40 (*v*:*v*). The final chl *a* molar concentration in the samples was 10^−5^ M. The concentration of dissolved oxygen in the solutions was 5.40 ± 0.58 mg/L.

### 3.2. Methods

#### 3.2.1. Stability

*Stability in the darkness*. The samples were stored in brown screw-cap glass vials, sealed with a cap with Teflon gasket, at room temperature, and remained in the dark. Each glass with the sample was opened only during taking the appropriate volume of solution to collect the absorption spectra and was immediately capped.

*Photostability.* The samples, inserted into a 1-cm-path-length quartz cuvette with a closed Teflon screw cap (Hellma, Müllheim, Germany), were illuminated with a halogen lamp (Bratek, Wrocław, Poland) under continuous stirring. The light power reaching the sample was approximately 0.1 mW/cm^2^ or 1 mW/cm^2^. The light intensity was measured using the HD 2302.0 light meter (Delta OHM, Caselle di Selvazzano, Italy). Temperature of the samples during illumination was stabilized in the range of 22–24 °C. It was controlled using a TES-1307 thermocouple probe (TES Electrical Electronic Corp., Taipei, Taiwan) placed directly into a sample.

#### 3.2.2. UV-Vis Absorption Spectroscopy

*UV-Vis absorption spectroscopy.* UV-Vis absorption spectra were registered using the Cary 60 (Agilent Technologies, Santa Clara, CA, USA) spectrophotometer in the range of 200–800 nm at room temperature.

#### 3.2.3. Steady-state Fluorescence Spectroscopy

Room temperature fluorescence emission spectra were recorded with a F-7000 spectrofluorometer (Hitachi, Tokyo, Japan) using a fluorescence cuvette with the dimensions of 5 × 5 mm^2^ (Hellma, Müllheim, Germany). The excitation was set at 434 nm. The excitation and emission slits were 5 nm.

#### 3.2.4. Dynamic Light Scattering

The micelle sizes were determined using Zetasizer Nano ZS90 (Malvern Instruments Ltd., Malvern, UK). The refraction index value was set at 1.33. The zeta potential was calculated according to the Smoluchowski equation.

### 3.3. Statistical Analysis

Statistical analysis was performed using Statistica13.1 (TIBCO Software Inc. Palo Alto, CA, USA) and OriginPro 2016 (OriginLab Co., Northampton, MA, USA) application. Data were expressed as the mean ± standard deviation (SD). Statistical comparisons were performed using ACNOVA to assess similarities within analyzed groups while time was considered covariate. The statistical significance of the differences between the groups was determined by covariance analysis, followed by comparison by the Tukey test (*p*-values < 0.05 were considered to be statistically significant).

## 4. Conclusions

In this paper, the stability of chl *a* monomers encapsulated into the CrEL nano-micelles was tested under dark and moderate light conditions. The dynamic light scattering method showed stability of the average size of CrEL micelles as chl *a* carriers even after 70 days storage in the dark. On the basis of the absorption spectra intensity, it was estimated that about the 50% of monomers pool was stable in the period of ~7 months in such a nano-system. The study utilizing steady-state fluorescence spectroscopy pointed out the negligible pool of the chl *a* destruction products in the samples which prove the high stability of the chl *a* monomeric form upon 5 wt% CrEL in the water medium along with time under the conditions indicated above. Moreover, the photostability of such a pigment-detergent system remained unaffected upon illumination with the visible light of 0.1 mW/cm^2^ power density for 1 h, typical of rooms with natural or artificial lighting. A lower photostability of chl *a* monomers in the CrEL micelles was noticed against photooxidation during 1-h irradiation with 1-mW/cm^2^ intensity. The overall results suggest the application of chl *a* monomers embedded in the CrEL micelles in a wide range of industrial and medical applications.

## Figures and Tables

**Table 1:** Significance of the power of light, exposition time and interactions based on the value of B_x_/Q_y_ for dark-adapted chl *a* and illuminated with white light for 60 min.

Effect Level	Mean Difference	Standard Error	t	*p*
1 mW	0.059150	0.011275	5.2460	0.000001 *
0.1 mW	−0.000048	0.011275	−0.0043	0.996596 ns
1 mW*Time	0.001410	0.000202	6.9916	0.000000 *
0.1 mW*Time	0.000224	0.000202	1.1093	0.269995 ns
Dark*Time	−0.000090	0.000247	−0.3659	0.715222 ns

* *p* < 0.05, ns = not significant.

## Supplementary Materials

The following are available online, Figure S1. Room temperature electronic absorption and emission fluorescence spectra measured from chl *a* dissolved in the 5% Cremophor EL nano-emulsion (CrEL micelles (5 wt%) + chl a; panels A, B) or 96% ethanol (EtOH+ chla *a*; panels C, D). The molar concentration of chl *a* in the samples was 10^−5^ M. The spectra were registered directly after the samples preparation (0 min, black solid line) and after 70 days storage of the samples in the dark (70 days, red dashed line). Figure S2. Room temperature absorption spectra of chl *a*. Chl *a* was dissolved in the 5% Cremophor EL nano-emulsion (CrEL micelles (5 wt%) + chl *a* (monomer), red solid line) or in the PBS buffer (PBS + chl *a* (aggregate), black, dashed line). The spectrum of chl *a* in the nano-micelles was measured after 70 days storage. The spectra were normalized in the Q_y_ band. Figure S3. Stability of chl *a* in the CrEL nano-micelles over time. Stability was expressed as the Q_y_ band intensity varying with time (70 days). Chl *a* at the molar concentration of 10^−5^ M was dissolved in the 5% Cremophor EL nano-emulsion (CrEL micelles (5 wt%) + chl *a*). The samples were stored in the dark for 70 days. The experiment was repeated three times (biological replicates). The presented mean values ±SD were obtained on the basis of the absorbance spectra. The value of half-lifetime (τ_1/2_) of chl *a* is included in the figure (obtained from the polynomial equation and estimated as the time during which a 50% decay of the Q_y_ band intensity takes place). Figure S4. The spectra of radiation emitted by the different light sources: halogen lamp used for illumination of chl *a* embedded in the CrEL nano-micelles (Panel A), natural sunlight (Panel B) and artificial light from the fluorescent bulb (Panel C). The spectra were detected using the optical emission spectrophotometer USB4000 (Ocean Optics). Figure S5. Panel A: (black solid line) the difference absorbance spectrum obtained from the original spectra presented in Figure 6, (red, dashed line) the difference absorbance spectrum obtained from the spectrum of chl *a* dissolved in the 5% Cremophor EL nano-emulsion (0 min) and the spectrum of the sample illuminated with red light (≥600 nm) for 60 min (60 min). Panel B: The fluorescence emission spectrum of chl *a* dissolved in the 5% Cremophor EL nano-emulsion (0 min, black solid line) and next illuminated with red light for 60 min (red, dashed line). The red-light power was 20 mW/cm^2^. The molar concentration of chl *a* in the samples was 10^−5^ M. Table S2. ACNOVA results. Table S2. Mean values of B_x_/Q_y_ ratio for time points and illuminations with standard deviations and corresponding post-hoc analysis results.
