# Supplementary material for: Stability of Chlorophyll a Monomer Incorporated into Cremophor EL Nano-Micelles under Dark and Moderate Light Conditions
Source: Molecules. 2020 Oct 30;25(21):5059. doi: 10.3390/molecules25215059 (PMC7672595; doi:10.3390/molecules25215059)
Supplement: Supplementary file 1 [file molecules-25-05059-s001.zip › molecules-969519-supplementary.pdf]

Supplementary materials

Stability of chlorophyll *a* monomer incorporated into Cremophor EL nano-micelles under dark and moderate light conditions

Ewa Janik-Zabrotowicz [1*], Marta Arczewska [2*], Patrycja Prochniewicz [1], Izabela Świetlicka [2], Konrad Terpilowski [3]

[1] Department of Cell Biology, Institute of Biological Sciences, Maria Curie-Sklodowska University, Akademicka 19, 20-033 Lublin, Poland, ewa.janik@poczta.umcs.lublin.pl

[2] Department of Biophysics, University of Life Sciences in Lublin, Akademicka 13, 20-950 Lublin, Poland, marta.arczewska@up.lublin.pl, izabela.swietlicka@up.lublin.pl

[3] Department of Physical Chemistry-Interfacial Phenomena, Maria Curie-Sklodowska University, Pl. Marii Curie-Sklodowskiej 3, 20–031 Lublin, Poland, terpil@poczta.umcs.lublin.pl

* Correspondence: ewa.janik@poczta.umcs.lublin.pl (E.J-Z.); marta.arczewska@up.lublin.pl (M.A.);
Tel./Fax: +48-81537-59-41 (E.J-Z.); +48-81445-69-05 (M.A.)

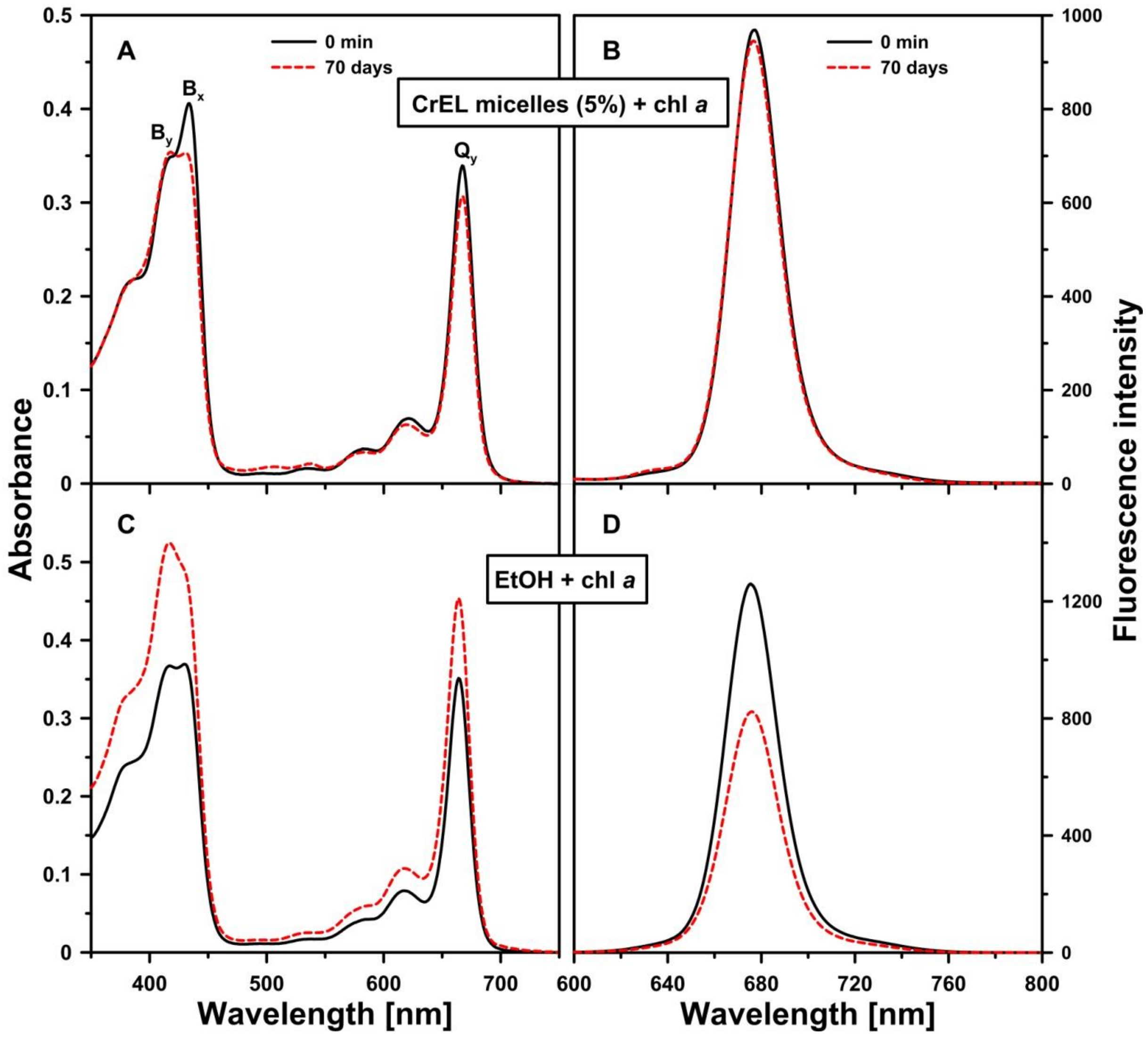

Fig. S1. Room temperature electronic absorption and emission fluorescence spectra measured from chl *a* dissolved in 5% Cremophor EL nano-emulsion (CrEL micelles (5wt%) + chl *a*; panels A, B) or 96% ethanol (EtOH+ chla *a*; panels C, D). The molar concentration of chl *a* in the samples was 10^{-5} M. The spectra were registered directly after the samples preparation (0 min, black solid line) and after 70 days storage of the samples in the dark (70 days, red dashed line). The experiment was repeated three times (biological replicates). The representative results are included in the figure.

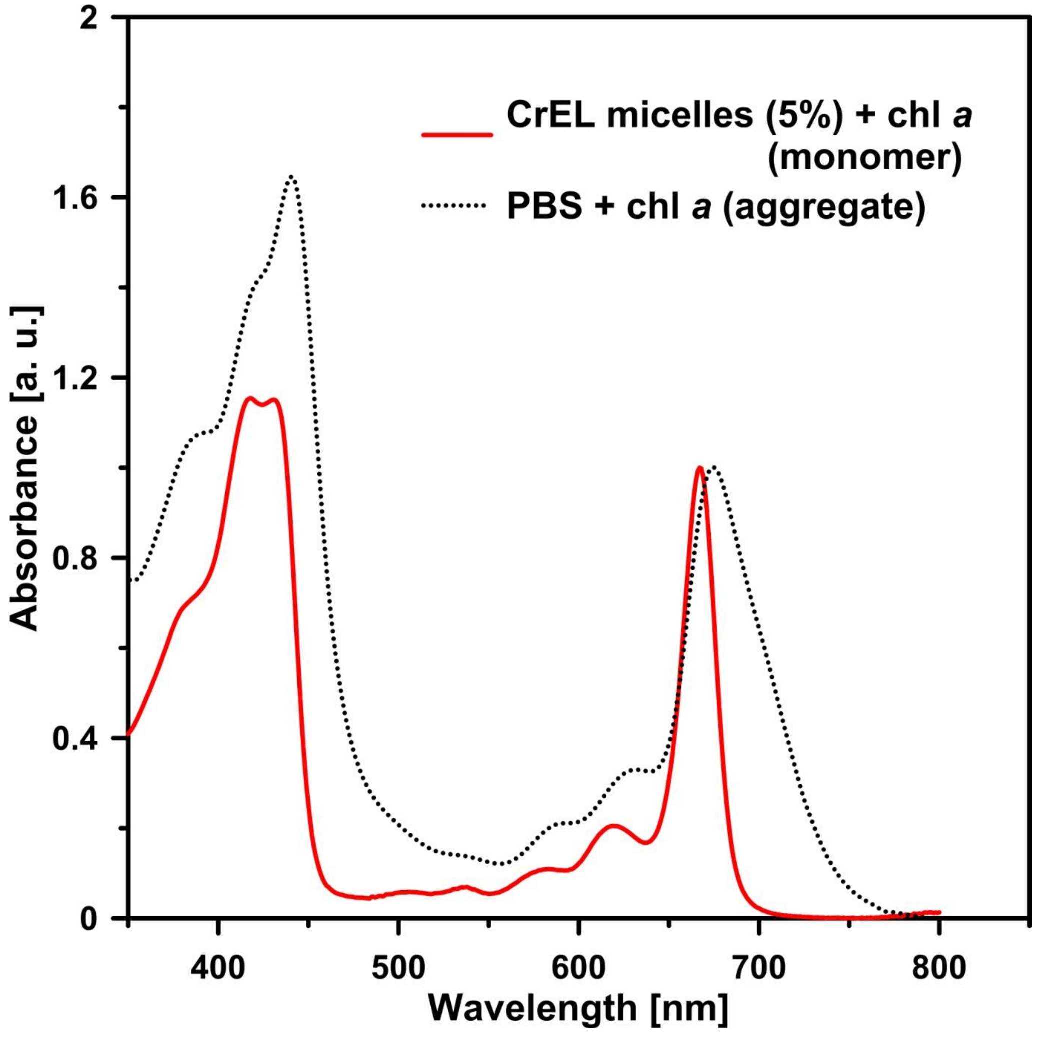

Fig. S2. The room temperature absorption spectra of chl *a*. Chl *a* was dissolved in 5% Cremophor EL nano-emulsion (CrEL micelles (5wt%) + chl *a* (monomer), red solid line) or PBS buffer (PBS+chl *a* (aggregate), black, dashed line). The spectrum of chl *a* in nano-micelles was measured after 70 days of the sample storage. The spectra were normalized in the Q_y band.

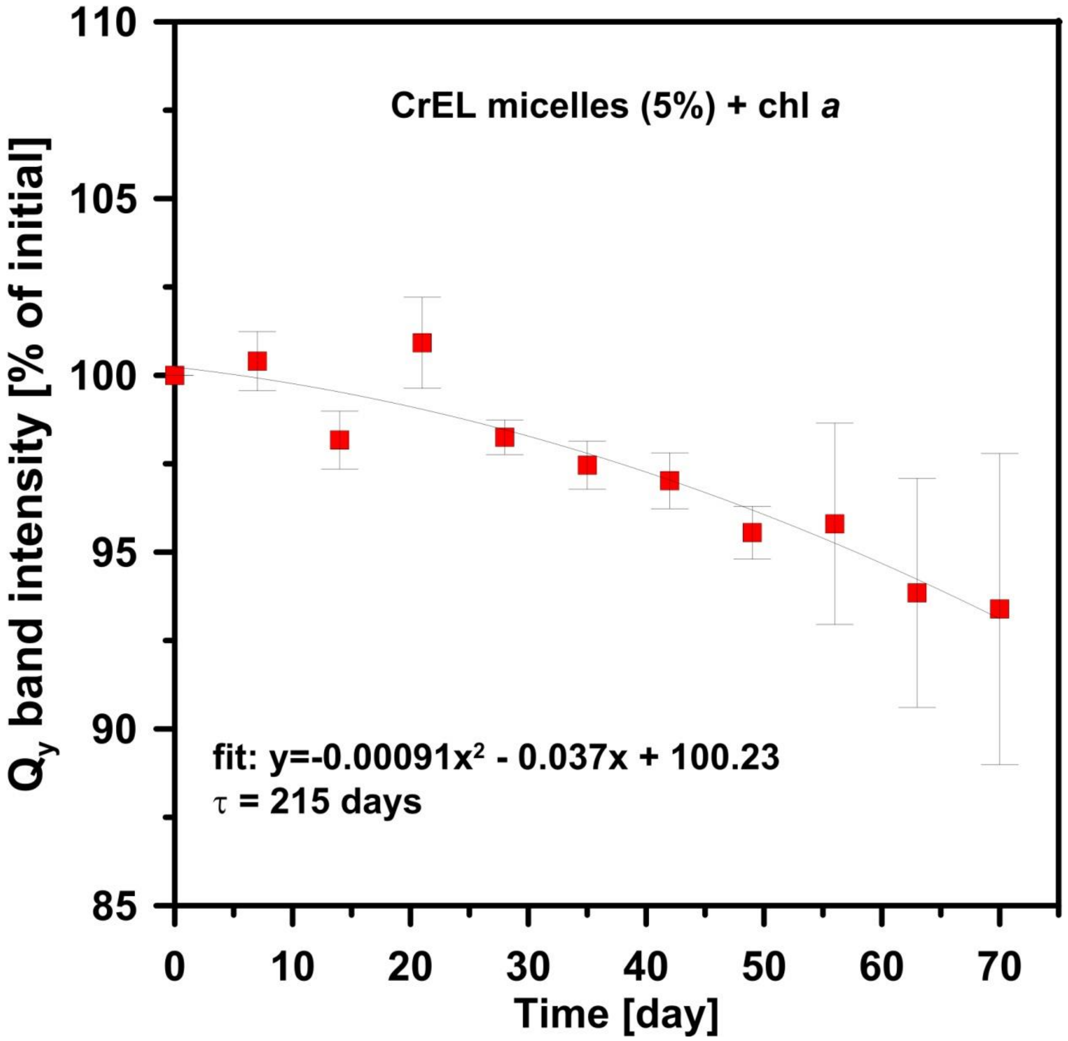

Fig. S3. Stability of chl *a* in CrEL nano-micelles over time. Stability was expressed as Q_y band intensity varying with time (70 days). Chl *a* at the molar concentration of 10^{-5} M was dissolved in 5% Cremophor EL nano-emulsion (CrEL micelles (5wt%) + chl *a*). The samples were stored in the dark for 70 days. The experiment was repeated three times (biological replicates). The presented mean values ±SD are obtained on the basis of the absorbance spectra. Value of half-lifetime ($\tau_{1/2}$) of chl *a* is included in the figure (obtained from the polynomial equation and estimated as the time during which a 50% decay of the Q_y band intensity take place).

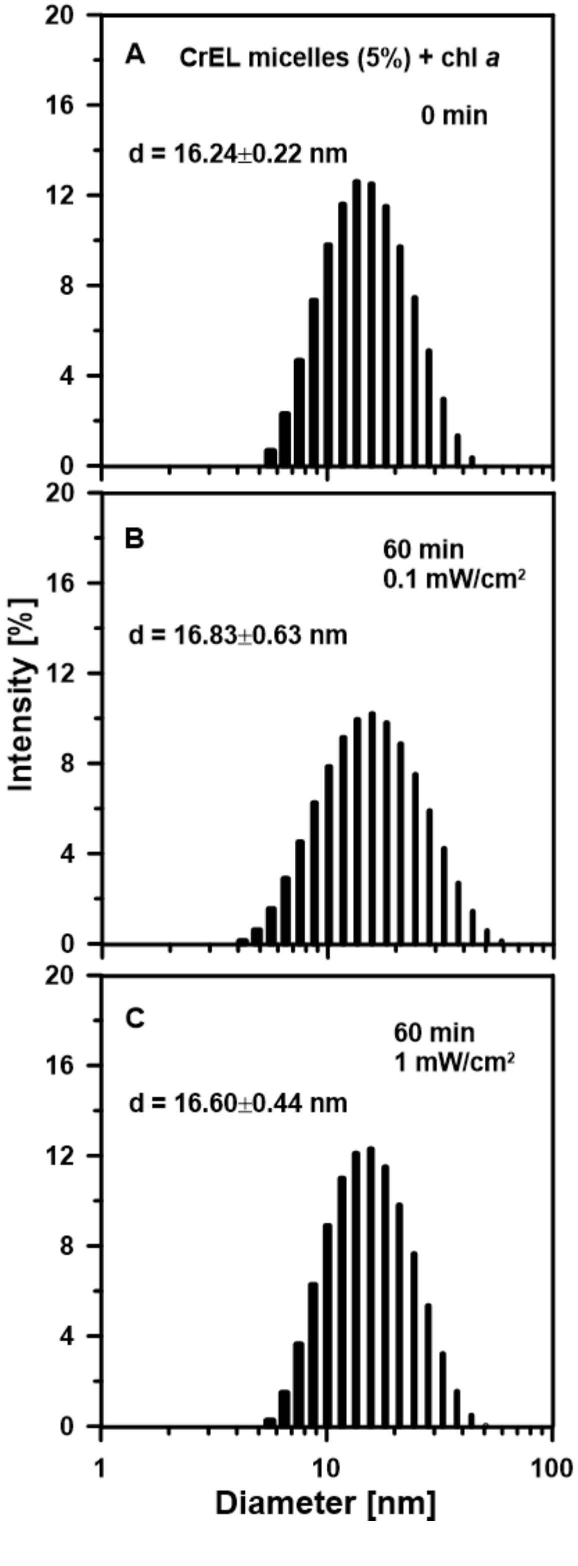

Fig. S4. The spectra of radiation emitted by the different light sources: halogen lamp used to illumination of chl a embedded in CrEL nano-micelles (Panel A), natural sunlight (Panel B), and artificial light from fluorescent bulb (Panel C). The spectra were detected using optical emission spectrophotometer (Ocean Optic).

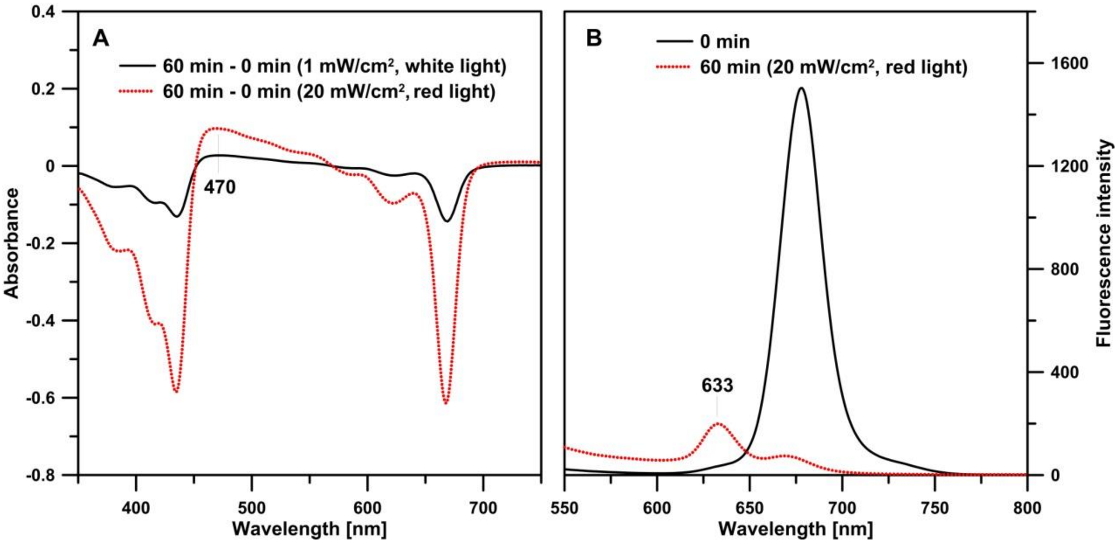

Fig. S5. Panel A: (black solid line) difference absorbance spectrum obtained from the original spectra presented in Figure 6, (red, dashed line) difference absorbance spectrum obtained from the spectrum of chl *a* dissolved in 5% Cremophor EL nano-emulsion (0 min) and the spectrum of the sample illuminated with red light (λ≥600 nm) for 60 min (60 min). Panel B: The fluorescence emission spectrum of chl *a* dissolved in 5% Cremophor EL nano-emulsion (0 min, black solid line) and next illuminated with red light for 60 min (red, dashed line). The red light power was 20 mW/cm^2. The molar concentration of chl *a* in the samples was 10^{-5} M.

Table S1. ACNOVA results.

	SS	df	MS	F	p
Constant	40.67229	1	40.67229	73243.49	0.000000
Illumination*Time	0.02790	3	0.00930	16.75	0.000000
Illumination	0.02390	2	0.01195	21.52	0.000000
Error	0.05442	98	0.00056		

Table S2. Mean values of B_x/Q_y ratio for time points and illuminations with standard deviations and corresponding post-hoc analysis results.

Time [min]	Illumination		
	Dark[a]	0.1[a] [mW/cm^2]	1[b] [mW/cm^2]
0	1.189 ±0.008	1.185 ±0.062	1.243 ±0.002
5	1.185 ±0.002	1.189 ±0.061	1.249 ±0.004
10	1.176 ±0.010	1.184 ±0.045	1.257 ±0.008
15	1.181 ±0.001	1.183 ±0.041	1.267 ±0.008
20	1.180 ±0.001	1.189 ±0.041	1.273 ±0.004
25	1.188 ±0.007	1.189 ±0.040	1.280 ±0.004
30	1.174 ±0.008	1.192 ±0.042	1.282 ±0.006
35	1.185 ±0.002	1.192 ±0.041	1.290 ±0.007
40	1.180 ±0.001	1.194 ±0.036	1.297 ±0.009
45	1.183 ±0.001	1.193 ±0.039	1.305 ±0.008
50	1.178 ±0.002	1.193 ±0.042	1.316 ±0.007
55	1.181 ±0.002	1.198 ±0.038	1.319 ±0.009
60	1.176 ±0.001	1.197 ±0.042	1.331 ±0.013

[a,b] – groups which are similar at $p < 0.05$.
